# IL-17F Induces CCL20 in Bronchial Epithelial Cells

**DOI:** 10.1155/2011/587204

**Published:** 2011-10-13

**Authors:** Kyoko Nozato, Junichi Fujita, Mio Kawaguchi, Gen Ohara, Yuko Morishima, Yukio Ishii, Shau-Ku Huang, Fumio Kokubu, Hiroaki Satoh, Nobuyuki Hizawa

**Affiliations:** ^1^Department of Respiratory Medicine, Institute of Clinical Medicine, University of Tsukuba, 1-1-1 Tennodai, Tsukuba, Ibaraki 3058575, Japan; ^2^Asthma and Allergy Center, Johns Hopkins University, Baltimore, MD 21224, USA; ^3^Department of Respiratory Medicine, Showa University Fujigaoka Hospital, Yokohama, Kanagawa 227-8501, Japan

## Abstract

IL-17F plays a crucial role in airway inflammatory diseases including asthma, but its function has not been fully elucidated. CCL20 is also involved in allergic airway inflammation, while its regulatory mechanisms remain to be defined. To further identify a novel role of IL-17F, the expression of CCL20 by IL-17F in bronchial epithelial cells and the signaling mechanisms involved were investigated. Bronchial epithelial cells were stimulated with IL-17F, and the levels of CCL20 gene and protein measured, with the effects of the addition of various kinase inhibitors and siRNAs also investigated. IL-17F significantly induced the expression of CCL20 gene and protein. Pretreatment with inhibitors for MEK1/2, Raf1 and MSK1, and overexpression of a Raf1 dominant-negative mutant significantly diminished IL-17F-induced CCL20 production. Moreover, transfection of the siRNAs targeting MSK1, p90RSK, and CREB blocked CCL20 expression. These findings suggest that IL-17F is able to induce CCL20 via Raf1-MEK1/2-ERK1/2-MSK1/p90RSK-CREB signaling pathway in bronchial epithelial cells. The IL-17F/CCL20 axis may be a novel pharmacological target for asthma.

## 1. Introduction

The IL-17 family of cytokines consists of six members, IL-17 (also called IL-17A), IL-17B, IL-17C, IL-17D, IL-17E (also called IL-25), and IL-17F [1–5]. We and other groups discovered human IL-17F [6–8]. We have reported that IL-17F is capable of inducing several cytokines and chemokines in bronchial epithelial cells [9–16]. The signaling pathway of IL-17F has been uncovered. Similar to IL-17A, the receptor for IL-17F is the heterodimeric complex of IL-17RA and IL-17RC [17]. Although human IL-17RA binds IL-17A effectively, it binds IL-17F with ~1000-fold lower affinity [18]. The relative binding affinity of IL-17F to IL-17RC is much stronger than to IL-17RA. Activation of the receptor by IL-17F leads to an interaction with Act-1 via the similar expression to fibroblast growth factor genes, IL-17 receptors, and TIR (SEFIR) domain [19]. This mediates activation of TNF receptor-associated factor (TRAF)-6 [19, 20]. Moreover, we have identified the downstream pathway of IL-17F receptor signaling. IL-17F activates the Raf1-MEK1/2-ERK1/2-MSK1/p90RSK-CREB signaling pathway [10–16]. In the airway of asthmatics, the expression of IL-17F is clearly upregulated [6], and is correlated with the disease severity [6, 21, 22]. We have also demonstrated that a coding-region variant (H161R) of the IL-17F gene is inversely associated with asthma and encodes an antagonist for the wild-type IL-17F [23, 24]. Moreover, a recent study showed that IL-17F has a possible role in the mechanism of steroid resistance in asthma [25]. These findings suggest that IL-17F is one of the important cytokines involved in the pathogenesis of allergic airway inflammation. IL-17F is derived from activated CD4^+^ T cells, basophils, and mast cells, three key-effector cell types involved in asthma [6]. Moreover, IL-17F is produced by a recently discovered lineage of CD4^+^ T cells, Th17 cells [26]. Th17 cells selectively produce hallmark cytokines IL-17A and IL-17F, but not IL-4 and INF*γ*, and they play a pivotal role in airway diseases including asthma [27]. In a mouse model of asthma, Th17 cells-mediated airway inflammation and airway hyperresponsiviness are steroid resistant [28]. In asthmatic patients, increased numbers of tissue-infiltrating Th17 cells are observed in the airway [29]. Another study demonstrated that the number of peripheral Th17 cells is significantly elevated in asthmatic patients compared with control subjects [30]. These findings suggest that Th17 cells have a potential role in the pathogenesis of asthma. However, it is unclear how Th17 cells traffic into the airway of asthmatics.

CCL20 is a CC chemokine and a unique functional ligand for CCR6. CCL20 is derived from bronchial epithelial cells in response to several stimuli such as proinflammatory cytokines, ambient particulate matter, and the proteolytic allergen Der p1 [31–33]. CCL20 is involved in the pathogenesis of airway inflammatory diseases including asthma. Indeed, its levels are significantly elevated in bronchoalveolar lavage (BAL) fluid from patients with allergic asthma when compared with control subjects [32]. It is also reported that the CCL20/CCR6 system plays a pivotal role in allergic airway responses such as airway resistance, airway eosinophilia, and production of IL-5 and IgE [34]. In addition, a recent study demonstrated that human Th17 cells predominantly express CCR6 [35]. This implies that CCL20 is able to attract Th17 cells into the site of airway inflammation via CCR6. However, inducers of epithelium-derived CCL20 in airway inflammation and its regulatory mechanisms have not been fully understood. To this end, the effects of IL-17F on expression of CCL20 were investigated. In this study, we demonstrated, for the first time, that IL-17F induces CCL20 in bronchial epithelial cells via the activation of RafI-MEK1/2-ERK1/2-MSK1/p90RSK-CREB signaling pathway.

## 2. Materials and Methods

### 2.1. Cell Culture

Two different bronchial epithelial cells were used in this study. A bronchial epithelial cell line, BEAS-2B, was cultured in Hanks' F12/DMEM (Biofluids, Rockville, Md, USA) with 10% heat-inactivated FBS, 100 U/mL penicillin, and 100 ng/mL streptomycin (Life Technologies-BRL, Gaithersburg, Md, USA). Normal human bronchial epithelial cells (NHBEs) were purchased from Lonza (Walkersville, Md, USA), and cultured in bronchial epithelial basal medium according to the manufacturer's instruction. The cells were cultured for no more than 3 passages prior to the analysis.

### 2.2. Analysis of CCL20 Gene Expression

Total RNA was extracted using RNeasy (Qiagen, Chatsworth, Caif, USA) from 1 × 10^6^ BEAS-2B cells at 4 hrs after stimulation with 10 and 100 ng/mL of IL-17F (R&D Systems, Minneapolis, Minn, USA). cDNAs were synthesized from 500 ng of total RNA using the cDNA synthesis kit (TOYOBO, Tokyo, Japan), followed by real-time PCR. The sequences of real-time PCR primers for CCL20: forward, 5′-CTGGCTGCTTTGATGTCAGT-3′, reverse, 5′-CGTGTGAAGCCCACAATAAA-3′; G3PDH: forward, 5′-ACCACAGTCCATGCCATCAC-3′, reverse, 5′-TCCACCACCCTGTTGCTGTA-3′. Real-time PCR was done using a SYBR Green PCR kit (Applied Biosystems, Tokyo, Japan), gene-specific primers, and an ABI 7700 thermal cycler. The gene expression levels for each amplicon were calculated using the ΔΔC_T_ method and normalized against G3PDH gene. The data were shown as fold induction relative to the control group. The values are expressed as mean ± SEM (*n* = 6 experiments).

### 2.3. Analysis of CCL20 Protein Expression

Cell supernatants in BEAS-2B cells and NHBEs were harvested from cultures in the absence or presence of 10 or 100 ng/mL of IL-17F at 2, 6, 12, 24, or 48 hrs after stimulation. Alternatively, BEAS-2B cells were also stimulated with 100 ng/mL of IL-17A and IL-17E (IL-25) (R&D Systems) for 24 hrs. CCL20 protein levels in the supernatants were determined with a commercially available ELISA kit (R&D Systems) according to the manufacturer's instruction. The values are expressed as mean ± SEM (*n* = 6 experiments).

### 2.4. Effect of Inhibitors on the Expression of CCL20

For analysis of involvement of the Raf1-MEK-ERK1/2-MSK1 pathway, BEAS-2B cells were treated in the presence or absence of the following kinase inhibitors at varying doses: MEK1/2 inhibitors, PD98059 (Calbiochem, La Jolla, Caif, USA), and U0126 (New England Bio Labs, Beverly, Mass, USA); p38MAPK inhibitor, SB202190 (Calbiochem); a Raf1 kinase inhibitor I (Calbiochem); a JNK inhibitor, SP600125 (Calbiochem); MSK1 inhibitors, H89 and Ro318220 (Calbiochem); and a vehicle control, DMSO (Me_2_SO) for 1 hr before treatment with IL-17F (100 ng/mL). The supernatants were harvested at 24 hrs after stimulation for analyses with ELISA. CCL20 protein levels in the supernatants were determined as described above. The values are expressed as mean ± SEM (*n* = 6 experiments). The total number of cells and cell viability at the end of the culture period for each experiment were similar among all culture conditions, as determined by trypan blue exclusion assay, suggesting that the inhibition of IL-17F-induced CCL20 expression did not result from cytotoxicity of those inhibitors (data not shown).

### 2.5. Overexpression of Dominant Negative Vector for Raf1 and Ras

The plasmids encoding pCMV-RafS621A Vector (dominant negative mutant of Raf-1) and pCMV-RasN17 Vector (dominant negative mutant of Ras) cloned into pCMV and a control vector were purchased from Clontech. The plasmids were prepared by using the Qiagen plasmid DNA preparation kit. Transfection experiments utilizing primary epithelial cells were technically difficult, and an epithelial cell line, BEAS-2B, was used instead. BEAS-2B cells were cultured in 100 mm plates and were transfected by Effectene Reagent (Qiagen) according to the manufacturer's instruction. The cells were selected with 500 *μ*g/mL of Geneticin (G418; Gibco/BRL). After selection, the cells were seeded into 6-well culture plates. The cells were near confluent, and the supernatants were then harvested at 24 hrs after stimulation with 100 ng/mL of IL-17F for analyses with ELISA. CCL20 protein levels in the supernatants were determined as described above. The values are expressed as mean ± SEM (*n* = 6 experiments).

### 2.6. Effect of Knockdown of p90RSK, MSK1, and CREB with siRNA

Pre-designed siRNAs for MSK1 (Bio Lad), p90RSK, CREB and, control siRNAs (Ambion, Tokyo, Japan) were used. The siRNA transfection into BEAS-2B cells was performed according to the manufacturer's instruction. The supernatants were then harvested at 24 hrs after stimulation with 100 ng/mL of IL-17F, and subjected for ELISA analyses, respectively (each *n* = 6 experiments). CCL20 protein levels in the supernatants are expressed as mean ± SEM.

### 2.7. Data Analysis

The statistical significance of differences was determined by analysis of variance (ANOVA). The values are expressed as mean ± SEM from independent experiments. Any difference with *P *values less than 0.05 was considered significant. When ANOVA indicated a significant difference, the Scheffe *F*-test was used to determine the difference between groups, since it is suitable for testing multiple comparisons.

## 3. Results

### 3.1. IL-17F Induces the Expression of CCL20

**Figure 1:**
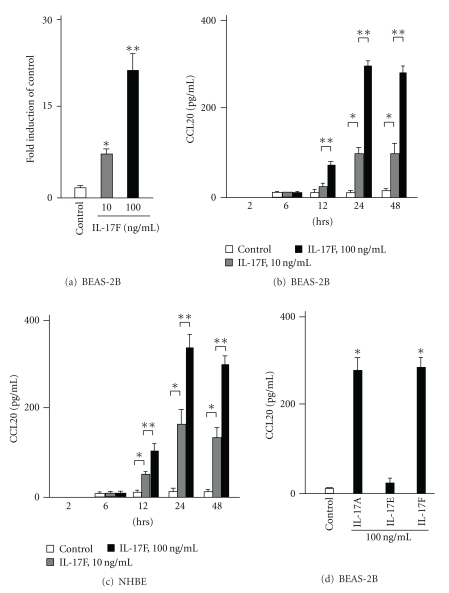
The expression of CCL20 gene and protein by IL-17F in bronchial epithelial cells. (a) CCL20 gene expression by real-time PCR in BEAS-2B cells. Real-time PCR was performed as described in Materials and Methods. BEAS-2B cells were stimulated with IL-17F for 4 hrs (*n* = 6). (c) CCL20 protein levels in supernatants in BEAS-2B cells. ELISA was performed (*n* = 6). (d) CCL20 protein levels in supernatants in NHBEs (*n* = 6). *P < .05 versus medium control. **P < .05 versus 10 ng/mL of IL-17F-stimulated cells. (e) CCL20 protein levels induced by IL-17A, IL-17E (IL-25), and IL-17F in supernatants in BEAS-2B cells (*n* = 6). *P < .05 versus medium control. The values are expressed as means ± SEM.

To examine whether IL-17F is able to induce CCL20 expression, BEAS-2B cells were stimulated with two doses of IL-17F. First, the levels of CCL20 gene expression were analyzed by real-time PCR. IL-17F significantly induced CCL20 gene expression in a dose dependent manner when compared with control (Figure 1(a)). Next, to analyze the protein expression for CCL20, the cells were cultured in the presence or absence of two different doses of IL-17F at five different time points. CCL20 proteins were not detected at the 2 hr time point and were weakly detected at the 6 hr time point. However, its protein levels in supernatants were significantly increased and peaked at the 24 hr time point (Figure 1(b)). Similarly, NHBEs also induced CCL20 expression in response to IL-17F and showed kinetics similar to those of BEAS-2B cells (Figure 1(c)). Another IL-17 family cytokine, IL-17A, showed similar potency in induction of CCL20 expression compared to IL-17F (Figure 1(d)). In contrast, IL-17E (IL-25) did not increase its expression in BEAS-2B cells.

### 3.2. MEK Inhibitors and Raf1 Kinase Inhibitor Inhibit IL-17F-Induced CCL20 Expression

**Figure 2:**
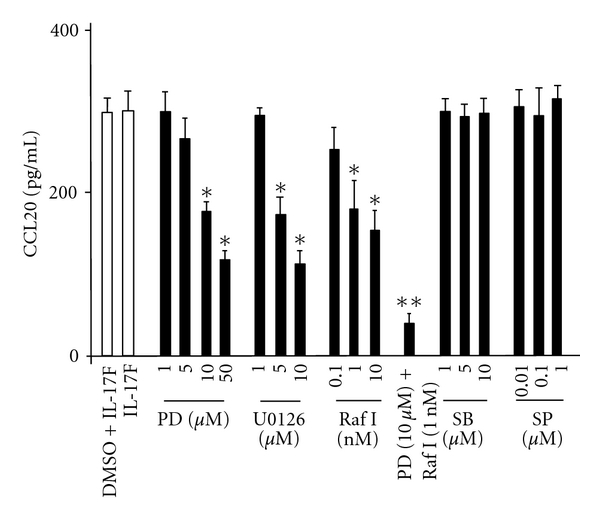
Effect of PD98059 (PD), U0126, Raf1 kinase inhibitor I (Raf I), SB202190 (SB), and SP600125 (SP) on CCL20 protein expression in BEAS-2Bs. The cells were pretreated for 1 hr as indicated before the 24-hr stimulation of IL-17F, and then CCL20 protein levels in supernatants were measured by ELISA. The values are expressed as means ± SEM (*n* = 6). *P < .05 versus IL-17F-stimulated cells in the absence of the inhibitor. **P < .05 versus the presence of individual inhibitor.

Pretreatment of the cells for 1 hr with each of the selective MEK inhibitors, PD98059 (10 and 50 *μ*M) and U0126 (5 and 10 *μ*M), and Raf1 kinase inhibitor I (1 and 10 nM) significantly decreased the levels of IL-17F-induced CCL20 expression in BEAS-2B cells, while 1 hr pretreatment of the cells with vehicle alone (0.1% DMSO) did not affect CCL20 expression. In addition, the protein levels of CCL20 were unchanged in IL-17F-treated cells in the presence of varying doses of a p38 MAPK inhibitor, SB202190, and a JNK inhibitor, SP600125 (Figure 2). While induction of CCL20 is partially inhibited by PD98059, U0126, or Raf1 kinase inhibitor I even at relatively high dose (50 *μ*M, 10 *μ*M and 10 nM, resp.), the combination of 10 *μ*M of PD98059 and 1 nM of Raf1 kinase inhibitor I inhibited, to a significant degree, the production of CCL20 (Figure 2).

### 3.3. Raf1 and Ras Dominant Negative Mutants Block IL-17F-Induced CCL20 Expression

**Figure 3:**
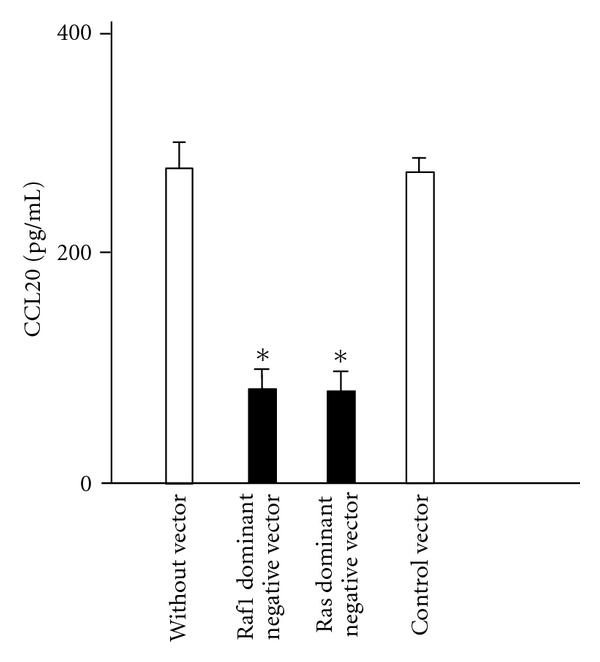
Effect of overexpression of dominant-negative mutants Raf1 and Ras on CCL20 protein expression in BEAS-2B cells. The cells overexpressing dominant-negative mutants were stimulated with IL-17F for 24 hrs, and CCL20 protein levels in supernatants were measured by ELISA. The values are expressed as means ± SEM (*n* = 6). *P < .05 versus IL-17F-stimulated cells without vector.

Overexpression of Raf1 and Ras dominant negative mutants in BEAS-2B cells significantly inhibited IL-17F-induced CCL20 expression (Figure 3), whereas the cells transfected with a control vector showed no significant effect in the level of CCL20 production.

### 3.4. MSK1 Inhibitors Inhibit IL-17F-Induced CCL20 Expression

**Figure 4:**
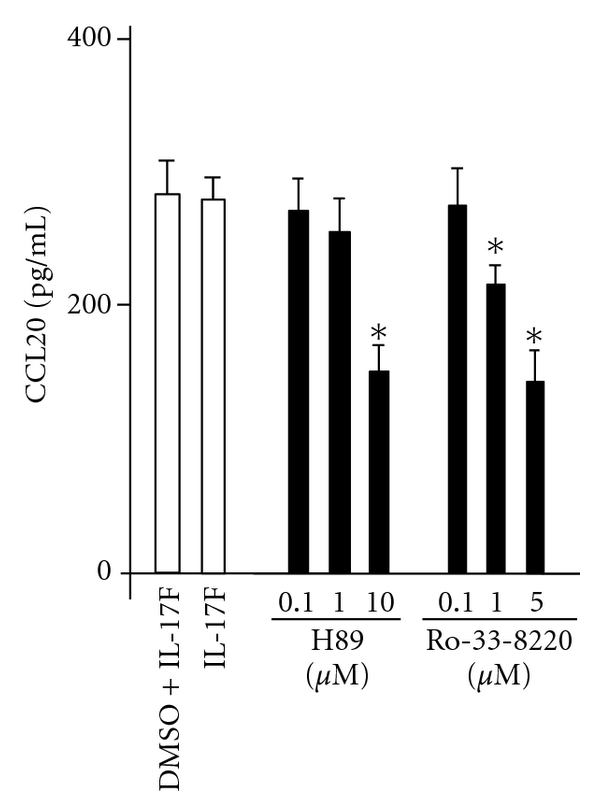
Effect of MSK1 inhibitors, H89, and Ro318220 on IL-17F-induced CCL20. BEAS-2B cells were pretreated for 1 hr as indicated before the 24-hr stimulation of IL-17F, and then CCL20 protein levels in supernatants were measured by ELISA. The values are expressed as means ± SEM (*n* = 6). *P < .05 versus IL-17F-stimulated cells in the absence of the inhibitor.

Next, to determine whether MSK1 affects IL-17F-induced CCL20 expression, the effects of MSK1 inhibitors were investigated. Pretreatment with two different MSK1 inhibitors, Ro-31-8220 and H89, significantly suppressed IL-17F-induced CCL20 expression (Figure 4).

### 3.5. siRNAs Targeting p90RSK, MSK1, and CREB Inhibit IL-17F-Induced CCL20 Expression

**Figure 5:**
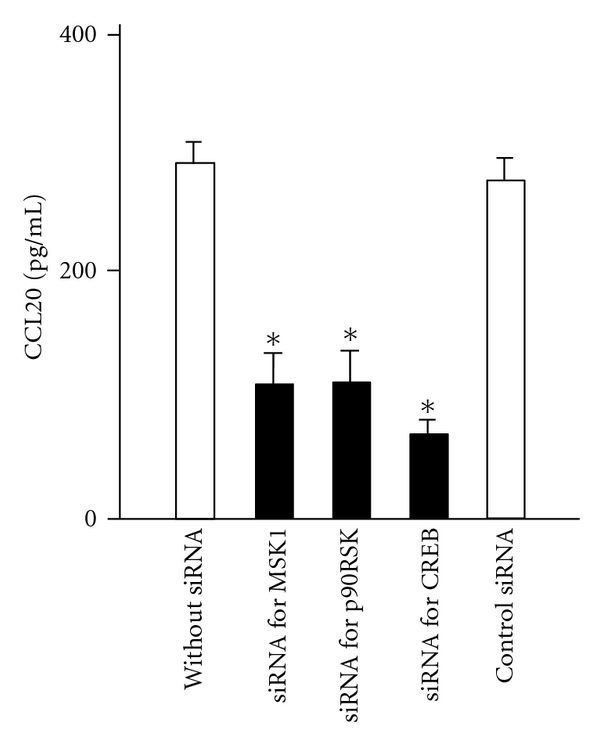
Effect of siRNA for p90RSK, MSK1 and CREB on IL-17F-induced CCL20 expression. BEAS-2B cells transfected with siRNAs as indicated were stimulated with IL-17F for 24 hrs, and then CCL20 protein levels in supernatants were measured by ELISA. The values are expressed as mean*s* ± SEM (*n* = 6). *P < .05 versus non-transfected cells.

Finally, the effect of siRNA targeting p90RSK, MSK1, and CREB on the induction of CCL20 expression by IL-17F was analyzed. As shown in Figure 5, its expression by IL-17F was significantly inhibited in cells transfected with siRNA targeting p90RSK, MSK1, and CREB, while no significant difference was seen in wild-type BEAS-2B cells and cells transfected with a control siRNA.

## 4. Discussion

In this paper, we demonstrated, for the first time, that IL-17F induces the expression of CCL20 in bronchial epithelial cells through the activation of the Raf1-MEK-ERK1/2-p90RSK/MSK1-CREB signaling pathway. These findings suggest that IL-17F is a potent inducer of CCL20, and the IL-17F/CCR20 axis may provide new insights into the pathophysiology of asthma.

IL-17F is potentially involved in the pathogenesis of asthma. Expression of the IL-17F gene is upregulated in BAL cells from asthmatics following segmental allergen challenge [6]. Its expression was seen in both bronchial epithelium and inflammatory infiltrates in asthmatic patients [21, 22]. Immunocytochemistry showed that IL-17F positive cells in the subepithelial component and epithelium are significantly elevated in severe asthma compared with control and mild asthmatic subjects [22]. Furthermore, a polymorphism in *IL-17F* gene that results in a loss of lung function mutation is inversely related to asthma risk [23, 24]. In the mouse model of asthma, IL-17F is clearly expressed in the lung [36] and is able to cause pulmonary neutrophilia and provides an additive effect on antigen-induced allergic inflammatory responses [37]. IL-17F exerts multiple functions. *In vitro*, we have demonstrated that IL-17F stimulates bronchial epithelial cells to induce numerous cytokines and chemokines such as IL-6, IL-8, IL-11, ENA-78, GRO*α*, GM-CSF, IP-10, and IGF-I [6, 9–16]. Furthermore, IL-17F is capable of inducing several cytokines and chemokines in eosinophils and lung structural cells including vein endothelial cells and fibroblasts [8, 38]. These cell types may play crucial roles in asthma in response to IL-17F. Prior to this study, it was unknown whether IL-17F affects CCL20 expression. In this paper, we have found, for the first time, that IL-17F is a potent inducer of CCL20 in bronchial epithelial cells. IL-17F shows the highest homology with IL-17A among the IL-17 cytokine family [6]. In this study, IL-17A is also able to induce CCL20 in bronchial epithelial cells, and this is consistent with the previous study [39]. Similarly to IL-17F, IL-17A produced CCL20 via the phosphorylation of ERK1/2, but not p38MAPK and JNK. Moreover, IL-17A activated NF-*κ*B as the downstream of ERK1/2. In contrast, we reported that IL-17F is not able to activate NF-*κ*B in bronchial epithelial cells [12]. At present, little is known about the difference of signaling pathway for IL-17A and IL-17F. Additional work is needed to determine their regulatory mechanisms.

CCL20 has a pivotal role in the pathogenesis of asthma, and is strongly derived from bronchial epithelial cells in response to a broad spectrum of asthma-related stimuli such as pro-inflammatory cytokines, ambient particulate matter, and the proteolytic allergen Der p1 [31–33]. In asthmatic patients, the level of CCL20 is significantly elevated in BAL fluid when compared with control subjects, and is more increased after endobronchial allergen provocation [32, 40]. Moreover, significant increase in its expression in BAL cells from subjects with corticosteroid-resistant asthma was seen when compared with those with corticosteroid-sensitive asthma [41]. In the mouse model of asthma, the CCL20/CCR6 system plays a pivotal role in allergic airway responses such as airway resistance, airway eosinophilia, and production of IL-5 and IgE [34]. Interestingly, CCR6 is predominantly expressed on human Th17 cells, and CCL20 shows chemotactic activity for Th17 cells [35, 42]. Emerging evidence suggests that Th17 cells are implicated in the pathogenesis of asthma [27]. Although little is known about how Th17 cells migrate into the airway, the current study suggests that IL-17F is able to attract Th17 cells into the site of airway inflammation via, at least partially, the CCL20/CCR6 system. Taken together, it is possible that IL-17F-induced epithelial CCL20 attracts Th17 cells into the airway, and accumulated Th17 cells establish a positive feedback loop resulting in the recruitment of additional Th17 cells via the inducing IL-17F. On the other hand, Th17 cells may not be the major cell source of IL-17F in airway diseases [43]. Indeed, IL-17F is produced by various cell types, such as basophils, mast cells, monocytes, memory CD4^+^ T cells, CD8^+^ T cells, *γδ*T cells, and NKT cells [6, 8, 44, 45]. Hence, these IL-17F-producing cells may exert an effect on bronchial epithelial cells to induce CCL20 and attract Th17 cells via CCL20/CCR6 system. The IL-17F/CCL20 axis might be especially important in the pathophysiologic events of allergic airway inflammation. However, further *in vivo* study is needed to clarify the importance of the IL-17F/CCL20 axis in asthma.

The signaling pathway of IL-17F has become clearer. We have previously demonstrated that the expression of IL-17F-induced cytokines and chemokines is dependent on the activation of ERK1/2, but not p38MAPK and JNK, in bronchial epithelial cells [9–13]. The signaling mechanisms of CCL20 expression are not yet fully understood. Here, we have identified, for the first time, that IL-17F-induced CCL20 expression is through the Raf1-MEK1/2-ERK1/2 pathway, since Raf1 kinase inhibitor and MEK1/2 inhibitors significantly decreased its expression. On the other hand, it is reported that CCL20 expression in bronchial epithelial cells is mediated by ERK1/2 and p38MAPK [31]. However, in this study, IL-17F-induced CCL20 expression is mediated by ERK1/2, but not p38MAPK, since p38MAPK inhibitor SB202190 did not affect its expression. This difference may be due to the stimuli used. Therefore, ERK1/2 may be a crucial signaling molecule for the IL-17F-induced CCL20 expression. These findings suggest that regulation of the Raf1-MEK1/2-ERK1/2 pathway may constitute a useful therapeutic target for IL-17F-associated diseases including asthma. In addition, we have recently identified that MSK1-CREB and p90RSK-CREB are two critical signaling pathways of IL-17F as the downstream elements of the Raf1-MEK1/2-ERK1/2 kinase cascade [14, 15]. These pathways are essential for CCL20 expression by IL-17F, since MSK1 inhibitors, Ro-31-8220 and H89, and siRNA targeting MSK1, p90RSK, and CREB significantly diminished its expression. Hence, CCL20 expression is mediated by the RafI-MEK1/2-ERK1/2-MSK1/p90RSK-CREB signaling pathway in the case of IL-17F in bronchial epithelial cells. These data suggest that this signaling pathway is a potential pharmacological target in the IL-17F-mediated airway inflammation. However, all inhibitors used in this study did not completely abrogate IL-17F-induced CCL20 expression. This suggests that the potential involvement of other signaling pathway. Further study is needed to clarify a novel signaling molecule of IL-17F.

In conclusion, we identified a novel function of IL-17F. IL-17F is capable of inducing CCL20 in bronchial epithelial cells, and its expression is mediated by the Raf1-MEK1/2-ERK1/2-MSK1/p90RSK-CREB signaling pathway. Taken together, the IL-17F/CCL20 axis may have an orchestrating role in the pathogenesis of asthma and could possibly provide a valuable therapeutic target for development of new strategies to treat asthma.

## Abbreviations

CREB: Cyclic AMP response element binding protein
ERK1/2: Extracellular signal-regulated kinase
MAPK: Mitogen-activated protein kinase
MEK: MAP kinase
MSK1: Mitogen- and stress-activated protein kinase1
NHBE: Normal human bronchial epithelial cell
p90RSK: p90 ribosomal S6 kinase.

## References

[1] Yao Z, Painter SL, Fanslow WC (1995). Human IL-17: a novel cytokine derived from T cells. *Journal of Immunology*.

[2] Shi Y, Ullrich SJ, Zhang J (2000). A novel cytokine receptor-ligand pair: identification, molecular characterization, and in vivo immunomodutory activity. *The Journal of Biological Chemistry*.

[3] Li H, Chen J, Huang A (2000). Cloning and characterization of IL-17B and IL-17C, two new members of the IL-17 cytokine family. *Proceedings of the National Academy of Sciences of the United States of America*.

[4] Starnes T, Broxmeyer HE, Robertson MJ, Hromas R (2002). Cutting edge: IL-17D, a novel member of the IL-17 family, stimulates cytokine production and inhibits hemopoiesis. *Journal of Immunology*.

[5] Lee J, Ho WH, Maruoka M (2001). IL-17E, a novel proinflammatory ligand for the IL-17 receptor homolog IL-17Rh1. *The Journal of Biological Chemistry*.

[6] Kawaguchi M, Onuchic LF, Li XD (2001). Identification of a novel cytokine, ML-1, and its expression in subjects with asthma. *Journal of Immunology*.

[7] Hymowitz SG, Filvaroff EH, Yin J (2001). IL-17s adopt a cystine knot fold: structure and activity of a novel cytokine, IL-17F, and implications for receptor binding. *The EMBO Journal*.

[8] Starnes T, Robertson MJ, Sledge G (2001). Cutting edge: IL-17F, a novel cytokine selectively expressed in activated T cells and monocytes, regulates angiogenesis and endothelial cell cytokine production. *Journal of Immunology*.

[9] Kawaguchi M, Adachi M, Oda N, Kokubu F, Huang SK (2004). IL-17 cytokine family. *Journal of Allergy and Clinical Immunology*.

[10] Kawaguchi M, Onuchic LF, Huang SK (2002). Activation of extracellular signal-regulated kinase (ERK)1/2, but not p38 and c-Jun N-terminal kinase, is involved in signaling of a novel cytokine, ML-1. *The Journal of Biological Chemistry*.

[11] Kawaguchi M, Kokubu F, Matsukura S (2003). Induction of C-X-C chemokines, growth-related oncogene *α* expression, and epithelial cell-derived neutrophil-activating protein-78 by ML-1 (Interleukin-17F) involves activation of Raf1-mitogen-activated protein kinase kinase-extracellular signal-regulated kinase 1/2 pathway. *Journal of Pharmacology and Experimental Therapeutics*.

[12] Kawaguchi M, Kokubu F, Odaka M (2004). Induction of granulocyte-macrophage colony-stimulating factor by a new cytokine, ML-1 (IL-17F), via Raf I-MEK-ERK pathway. *Journal of Allergy and Clinical Immunology*.

[13] Kawaguchi M, Adachi M, Huang SK (2003). Structural and functional analysis of a new cytokine, ML-1 (interleukin-17F). *Allergology International*.

[14] Kawaguchi M, Kokubu F, Huang SK (2007). The IL-17F signaling pathway is involved in the induction of IFN-*γ*-inducible protein 10 in bronchial epithelial cells. *Journal of Allergy and Clinical Immunology*.

[15] Kawaguchi M, Fujita J, Kokubu F (2009). IL-17F-induced IL-11 release in bronchial epithelial cells via MSK1-CREB pathway. *American Journal of Physiology*.

[16] Kawaguchi M, Fujita J, Kokubu F (2010). Induction of insulin-like growth factor-I by interleukin-17F in bronchial epithelial cells. *Clinical and Experimental Allergy*.

[17] Toy D, Kugler D, Wolfson M (2006). Cutting edge: interleukin 17 signals through a heteromeric receptor complex. *Journal of Immunology*.

[18] Kuestner RE, Taft DW, Haran A (2007). Identification of the IL-17 receptor related molecule IL-17RC as the receptor for IL-17F. *Journal of Immunology*.

[19] Yang XO, Seon HC, Park H (2008). Regulation of inflammatory responses by IL-17F. *Journal of Experimental Medicine*.

[20] Rong Z, Cheng L, Ren Y (2007). Interleukin-17F signaling requires ubiquitination of interleukin-17 receptor via TRAF6. *Cellular Signalling*.

[21] Kokubu F, Matsukura S, Kawaguchi M, Osakabe Y (2008). Respiratory viruses and bronchial asthma. *Japanese Journal of Allergology*.

[22] Al-Ramli W, Préfontaine D, Chouiali F (2009). TH17-associated cytokines (IL-17A and IL-17F) in severe asthma. *Journal of Allergy and Clinical Immunology*.

[23] Kawaguchi M, Takahashi D, Hizawa N (2006). IL-17F sequence variant (His161Arg) is associated with protection against asthma and antagonizes wild-type IL-17F activity. *Journal of Allergy and Clinical Immunology*.

[24] Hizawa N, Kawaguchi M, Huang SK, Nishimura M (2006). Role of interleukin-17F in chronic inflammatory and allergic lung disease. *Clinical and Experimental Allergy*.

[25] Vazquez-Tello A, Semlali A, Chakir J (2010). Induction of glucocorticoid receptor-*β* expression in epithelial cells of asthmatic airways by T-helper type 17 cytokines. *Clinical and Experimental Allergy*.

[26] Harrington LE, Hatton RD, Mangan PR (2005). Interleukin 17-producing CD4+ effector T cells develop via a lineage distinct from the T helper type 1 and 2 lineages. *Nature Immunology*.

[27] Alcorn JF, Crowe CR, Kolls JK (2009). TH17 cells in asthma and COPD. *Annual Review of Physiology*.

[28] McKinley L, Alcorn JF, Peterson A (2008). TH17 cells mediate steroid-resistant airway inflammation and airway hyperresponsiveness in mice. *Journal of Immunology*.

[29] Pène J, Chevalier S, Preisser L (2008). Chronically inflamed human tissues are infiltrated by highly differentiated Th17 lymphocytes. *Journal of Immunology*.

[30] Wong CK, Lun SWM, Ko FWS (2009). Activation of peripheral Th17 lymphocytes in patients with asthma. *Immunological Investigations*.

[31] Reibman J, Hsu Y, Chen LC, Bleck B, Gordon T (2003). Airway epithelial cells release MIP-3*α*/CCL20 in response to cytokines and ambient particulate matter. *American Journal of Respiratory Cell and Molecular Biology*.

[32] Pichavant M, Charbonnier AS, Taront S (2005). Asthmatic bronchial epithelium activated by the proteolytic allergen Der p 1 increases selective dendritic cell recruitment. *Journal of Allergy and Clinical Immunology*.

[33] Starner TD, Barker CK, Jia HP, Kang Y, McCray PB (2003). CCL20 is an inducible product of human airway epithelia with innate immune properties. *American Journal of Respiratory Cell and Molecular Biology*.

[34] Lukacs NW, Prosser DM, Wiekowski M, Lira SA, Cook DN (2001). Requirement for the chemokine receptor CCR6 in allergic pulmonary inflammation. *Journal of Experimental Medicine*.

[35] Annunziato F, Cosmi L, Santarlasci V (2007). Phenotypic and functional features of human Th17 cells. *Journal of Experimental Medicine*.

[36] Suzuki S, Kokubu F, Kawaguchi M (2007). Expression of interleukin-17F in a mouse model of allergic asthma. *International Archives of Allergy and Immunology*.

[37] Oda N, Canelos PB, Essayan DM, Plunkett BA, Myers AC, Huang SK (2005). Interleukin-17F induces pulmonary neutrophilia and amplifies antigen-induced allergic response. *American Journal of Respiratory and Critical Care Medicine*.

[38] Cheung PFY, Wong CK, Lam CWK (2008). Molecular mechanisms of cytokine and chemokine release from eosinophils activated by IL-17A, IL-17F, and IL-23: implication for Th17 lymphocytes-mediated allergic inflammation. *Journal of Immunology*.

[39] Kao CY, Huang F, Chen Y (2005). Up-regulation of CC chemokine ligand 20 expression in human airway epithelium by IL-17 through a JAK-independent but MEK/NF-*κ*B-dependent signaling pathway. *Journal of Immunology*.

[40] Goleva E, Hauk PJ, Hall CF (2008). Corticosteroid-resistant asthma is associated with classical antimicrobial activation of airway macrophages. *Journal of Allergy and Clinical Immunology*.

[41] Francis JN, Sabroe I, Lloyd CM, Durham SR, Till SJ (2008). Elevated CCR6+ CD4+ T lymphocytes in tissue compared with blood and induction of CCL20 during the asthmatic late response. *Clinical and Experimental Immunology*.

[42] Hirota K, Yoshitomi H, Hashimoto M (2007). Preferential recruitment of CCR6-expressing Th17 cells to inflamed joints via CCL20 in rheumatoid arthritis and its animal model. *Journal of Experimental Medicine*.

[43] Prause O, Bossios A, Silverpil E (2009). IL-17-producing T lymphocytes in lung tissue and in the bronchoalveolar spaceafter exposure to endotoxin from Escherichia coli in vivo—effects of anti-inflammatory pharmacotherapy. *Pulmonary Pharmacology and Therapeutics*.

[44] Yang XO, Nurieva R, Martinez GJ (2008). Molecular antagonism and plasticity of regulatory and inflammatory T cell programs. *Immunity*.

[45] Ciric B, El-behi M, Cabrera R, Zhang GX, Rostami A (2009). IL-23 drives pathogenic IL-17-producing CD8+ T cells. *Journal of Immunology*.

